# Why seek a second consultation at an emergency centre? A qualitative study

**DOI:** 10.4102/phcfm.v9i1.1397

**Published:** 2017-07-27

**Authors:** Lize Crafford, Louis S. Jenkins

**Affiliations:** 1 Division of Family Medicine and Primary Care, Faculty of Medicine and Health Sciences, University of Stellenbosch, South Africa

## Abstract

**Background:**

The inappropriate use of emergency centres (ECs) is an expanding problem globally. The high attendance of non-urgent return presentations to ECs is recognised as part of the problem, placing an unnecessary demand on limited staff and resources. Of unscheduled returns 34% of cases had no change to diagnosis or treatment with the conclusion that 80% of re-attendance could be attributed to deficiencies in the initial consultation. This study aimed to evaluate the reasons why patients sought an early second consultation for the same complaint at a hospital EC in South Africa, by exploring the patient’s experience and shortcomings in the first consultation.

**Method:**

A qualitative study was conducted using in-depth, semi-structured interviews with 20 purposively selected participants who presented to a rural regional provincial hospital’s EC within 7 days of a prior consultation for the same complaint. Verbatim transcripts were analysed using the framework method.

**Results:**

The main reasons for a second consultation were symptom related factors and the need for diagnostic certainty. The major themes around patient experience of the initial consultation were shortcomings in effective evaluation and management of pain, diagnostic uncertainty including poor examination, poor explanation, uncertain access and follow-up and societal encouragement to utilise a hospital EC.

**Conclusion:**

Further interventions should explore pain as a presenting symptom of the illness experience, and promote competence in addressing physical and psychological causative factors within a patient-centred approach for all health staff, especially in primary care services.

## Background

Globally, the increased utilisation of emergency centres (ECs) is an acknowledged problem in health care systems. This has not only led to crowding of units, prolonged waiting periods and delays to treatment, but also to patient dissatisfaction and reduced productivity of health care workers.^1,2,3^ Factors contributing to this increase of EC usage have been shown to be the lack of health care insurance, poor access to primary care, the increase in communicable diseases, inappropriate EC use for non-urgent complaints by patients and the return of patients to the ECs.^3,4^

Inappropriate use of ECs by non-urgent patients has been found to be because of convenience, accessibility of facilities and resources, diagnostics, costs and dissatisfaction with caregivers in primary care.^4,5,6^ This explains the phenomena of patients who identify ECs as their source of primary care, leading to repeated visits with non-urgent complaints that burden the health systems with increased expenses compared to that of clinic follow-up.^2,3^

Internationally the rate of unscheduled return visits within 72 h of the initial consultation is between 2.0% and 3.6%.^2,7,8,9^ Although the number of patient returns is small, they do add to overcrowding. Many of these unscheduled returns have been shown to be unnecessary, non-urgent and not in need of emergency care.^2,3^ The main reasons include persisting symptoms, progression of disease and complication of treatment in 47.9% to 87.0% of cases.^4,9,10,11,12^ To a lesser degree studies have identified patient-related factors attributing to unscheduled returns and suggested this as an area of intervention.^9,10,13^ Thirty-four per cent of returns for the same complaint had no change to diagnosis or treatment and up to 80.0% of re-attendance is attributed to deficiencies in the initial consultation.^13^

The urge for a second opinion is driven by either internal motives such as the need for reassurance, information and certainty or by an external motive, particularly the negative appraisal of the first consultation.^14,15^ Dissatisfaction is a multi-factorial concept involving elements of too little consultation time, the need for better explanation, communication and shared decision-making.^14^ Patient perceptions have been shown to predict outcomes of consumer satisfaction and are recognised as an acceptable measurement of service quality.^16,17^

South Africa is a resource-poor, developing country, where overcrowding of public ECs is commonly experienced, with critical consequences.^5,18^ The four-fold burden of disease, which includes HIV and TB, non-communicable diseases, violence and maternal and child illnesses, compounded by mental illness and substance abuse, often overwhelms the public primary health care service and spills over into hospital ECs.^19^ The majority of South Africans do not have health insurance and make use of the public health system, where primary health care is free and nurse-driven. Many of these patients also pay out of pocket to utilise the private health system. About 20% of South Africans do have health insurance and make use of the private health system of general practitioners (GPs) and specialists.

The *National Health Act of 2003* emphasises person-centred health care, while the Western Cape 2030 Health Plan’s vision is person-centred quality care with the focus on patient experience of health care as first principle.^20,21^ Previous work performed in Eden showed that 65.0% of patients presenting after hours to the hospital EC had routine non-urgent complaints, of which 47.0% required primary level care.^18^ Becker found that 88.9% of George hospital EC users were self-referred of which only 4.9% were considered appropriate for EC care.^5^ Understanding patients’ reasons to seek a second consultation and exploring their experience of the initial consultation are fundamental to address unscheduled returns.

The aim of this study was to explore patients’ experience of their first consultation and any shortcomings that led to seeking an early second consultation at a rural regional hospital EC. We were specifically interested in the main reason for the second consultation, the socio demographic, logistical and personal attributes of the patients seeking a second opinion and the possible shortcomings of the first consultation.

## Methods

### Study design

This was a qualitative study using in-depth, semi-structured interviews. Qualitative research was chosen as this was deemed the best way to obtain a deep understanding of patients’ experience of the initial consultation and reasons for seeking a second consultation.^16,17^

### Study setting

The study was conducted in the EC of George provincial hospital, a rural regional state hospital in the Eden district of the Western Cape province of South Africa. It is the only public regional hospital in the Eden and Central Karoo districts and provides a higher level of care for 10 surrounding primary health care clinics (PHCs) and 10 district hospitals. The EC attends to approximately 3400 patients monthly. No referral is necessary to access the emergency services and being the only public health facility open after hours in the George sub-district, all patients, including those with less urgent complaints, utilise this service.

### Sampling and data collection

Study participants were purposively selected from all adult (18 years of age or older) patients presenting to the EC during working hours between 08:00 and 16:00, during the month of October 2014.^22^ Inclusion criteria were: adults, 18 years and older, who presented within 7 days of a prior consultation for the same complaint at any health facility, who was not referred or scheduled for a follow-up, who could converse comfortably in Afrikaans or English and who was not clinically unstable. Prior consultations included a consultation at a primary health care facility, private GP or any EC. The mean time period for returns for a related complaint has been found to be 4.5 days, and 7 days is advocated to capture all data of related returns.^8^

The researcher approached patients during their triage procedure in the EC with the question, ‘Have you visited a health care practitioner within the last seven days for the same complaint?’ Eligible patients were consulted in private and the aim and objectives of the study explained to them. Patients who agreed to participate proceeded to sign an informed consent form. In-depth, semi-structured interviews, as well as demographic questionnaires, were conducted with each participant in a private room within the EC. Twenty-one participants were interviewed by the researcher, with interviews lasting up to 25 min each. An interview guide was used and volunteered themes were further explored. The interviews were audio taped and field notes were made by the researcher. One interview was not included as the interview was discontinued as a result of language barriers.

Continued discussion with a senior co-researcher during the interview phase led to revision of the interview guide, formulating extended questions to further explore prominent themes and research objectives. The interviews stopped when a point of information saturation was reached. Verbatim transcription of the audio recordings was performed.

### Analysis

Twenty audio recordings, verbatim transcriptions, demographic questionnaires and the reflective field notes of the researcher were analysed using the framework method.^23,24^ After familiarisation, themes were inductively identified. Thereafter an index of numerical codes was assigned to the themes in the transcripts, and finally a chart of major and minor themes was developed, with interpretation of contradictions and associations between major and minor themes explored.^24^

To improve credibility, the data from the interviews were triangulated with findings in the literature and by involving a co-researcher in the analysis. The discussion of themes and interpretation of data between two independent researchers helped to limit bias. Confirmability and objectivity was sought by inclusion of a reflexive paragraph.

### The role of the researcher

Personal reflexivity of the self as research tool to acknowledge the impact of her education, Caucasian heritage, privileged upbringing and lived experience as a doctor working in the EC of George hospital needed to be explored to caution against pre-judgemental assumption regarding the study question and objectives. The researcher attempted to limit biases by carefully constructing open-ended questions in the interview guide, together with the co-researcher who encouraged spontaneous discussion of topics and themes. She was aware that the doctor title and being a female, White student from the university tended to position her in a ‘patriarchal’ role relative to the participants. Although introduced by her first name with the explanation that she is a postgraduate student who used to work in the EC before, it was clear that the participants still associated her with the institution and the larger health system. The researcher had to consciously refrain from the directive, closed question interview style taught during undergraduate studies. She was taught to be a reductionist detective of symptoms and signs and to focus on the search for the most likely diagnosis, as opposed to a more constructivist approach of allowing for ‘new truths’ to emerge from another’s world view.

Interpersonal reflexivity was sought in compiling demographic outlines of the participants and exploring cues from open-ended answers to better understand their lived experiences. The researcher found it difficult to put down the doctor’s detective magnifying glass in search of presumed theories as stated in previous literature. As she matured in her interview style and reassessed what she brought to the equation, she learnt how to allow the participants to truthfully explain their experiences. Soon it became more natural to walk with them through their memories and try to observe their reality. The search for the assumed correct answers faded and she understood that she needed to listen to the spoken and unspoken words to make sense of their lived experiences. Being able to converse with them in Afrikaans and English improved the limitations as would have been set by using an interpreter. The researcher kept a reflexive journal of all interviews to alert herself to her own feelings, emotions and concerns during the interviews, and discussed these with the co-researcher, to allow for perspective.

### Ethical consideration

Ethics approval was obtained from the University of Stellenbosch Higher Research Ethics Committee (Nr S13/07/126), the Western Cape provincial health department and George Provincial Hospital.

## Results

Twenty participants, 9 female and 11 male, between the ages of 20 and 63 years (mean 42.6 years), fulfilled the inclusion criteria and participated. Seventy per cent of the participants’ first language was Afrikaans, 25% isiXhosa and 5% English, which is a true reflection of the Eden district demographics (70.8% Afrikaans, 18.3% isiXhosa, 7.5% English).^25^ Thirty-five per cent of participants relied on public transport to access health care. Two participants bypassed the primary health care services by utilising ambulance transport to the hospital EC. Eleven participants’ first consultations were at their local PHC, seven presented themselves to the EC after a prior visit and only two participants sought medical care at a private GP. The distance from home to the EC ranged from 2.5 km to 23.0 km.

Six major themes with closely connected minor themes emerged from the interviews (Table 1).

**TABLE 1:** Major and minor themes.

No.	Major themes	Minor themes
1.	I expect the pain to be stilled	Receiving only pills for pain
2.	They cannot tell you what is going on inside	Doctor controls access to investigations
3.	You do not get a real examination	Poor explanation
4.	Uncertain follow-up	Doubting whether it would help
5.	I did not know whether they were going to help me	Told to come back another day
6.	They told me I must rather go to the hospital	

### I expect the pain to be stilled

The majority of participants reflected that ineffective pain relief was the single most important factor motivating a second consultation. The pursuit of adequate pain management drove them to the hospital EC.

‘I expect the pain to be stilled, if he [*the pain*] can only disappear or become lighter then it will be acceptable to me. … That is what I really want. It is the pain that pushed me to the hospital.’ (Participant 11, male, 39 years)

Closely linked with the expectation of effective pain relief was the minor theme of general dissatisfaction with the receiving of ‘only pills’ for the pain. Participants were looking for a diagnosis with an effective management plan and expected more than merely the routine prescription of pills that they received at their initial consultation.

‘I thought that they would do further investigations to look where the pain is coming from, but nothing of that.’ (Participant 12, male, 27 years)

‘I think they could have given me an injection.’ (Participant 3, female, 53 years)

They perceived this as an inability of health care workers to adequately diagnose and manage their pain, which led to distrust and a second consultation. This emerged from participants whose first consultations were at the primary health clinic, hospital EC and private GPs.

‘They [*PHC sister*] can’t even tell me or what [*is wrong*]. … Then she gave me pills … she again gave me pills.’ (Participant 16, male, 20 years)

‘Then he [*GP*] took my arm … and he just checked me and he gave me some pain killers.’ (Participant 19, female, 38 years)

‘It doesn’t help you get pills from the clinic repeatedly.’ (Participant 2, female, 60 years)

### They cannot tell you what is going on inside

A second theme that repeatedly emerged, and related to the first one, was the need to find a doctor who could ‘look inside’ to see what is really wrong, thereby finding diagnostic certainty.

‘At the clinic they also check your blood pressure and everything, but they can’t tell you what the problem truly is … what is going on inside.’ (Participant 18, female, 57 years)

‘She [*doctor*] didn’t just examine me and made her own conclusion. The technology that was produced by the machine today, told her what is going on inside my body.’ (Participant 5, female, 43 years)

They felt that at the hospital EC the technology available would allow the doctors to ‘look inside’ and find the cause of their illness. Most participants were limited to the public sector because of financial constraints.

‘Actually it is easier to come to hospital … because see, here you go under machines.’ (Participant 14, female, 49 years)

‘No, it is not a must that I had to come here [*EC*], but see here they at least take X-rays and with that I’m satisfied.’ (Participant 7, male, 49 years)

The closely connected minor theme of the doctor holding the power to diagnose by controlling access to investigations made the participants feel disempowered, which resulted in many decisions about their health being handed over to the health care providers. They perceived that the doctor held the power that determined access to technology that would unlock the cause and cure of their pain.

‘This [*health care access*] is a problem and if you talk about it, you are always the one in the wrong. … Keep quiet and be the least.’ (Participant 1, female, 20 years)

‘Now okay, who am I to complain? Because the doctor’s word is above my word.’ (Participant 8, male, 63 years)

‘Because I can’t follow my own way, I need to get permission from the doctor.’ (Participant 7, male, 49 years)

At times these investigation requests seemed inappropriate as participant 15 persisted when asked what he wanted from his second consultation: ‘They need to check the pain … they need to check it with the scanner [*CT scan*]’.

Patient disempowerment was strengthened by the primary health care providers at the clinics who used limited resources as the reason to justify their lack of diagnostic certainty:

‘Then she [*sister*] said, they [*PHC*] don’t have the facilities to see what the exact problem is.’ (Participant 1, female, 20 years)

The expectation of special investigations often stemmed from discussions in the initial consultation. Such was the case with participant 17, who was told at her first consultation that she ‘might’ need an ultrasound.

‘So I decided to come back again. Maybe he must do the sonar thing, so that they can be sure what it is.’ (Participant 17, female, 34 years)

‘But now I will go to the hospital, because maybe they can do something for me to see exactly what the problem is.’ (Participant 1, female, 20 years)

### You do not get a real examination

Some participants expressed unhappiness with the clinical examination of the clinical nurse practitioners at their local clinic, expecting that a doctor would do better:

‘At the end you only see a sister, and from the sister you only get Panado. You don’t get a real examination. … She will not be like a doctor.’ (Participant 2, female, 60 years)

‘I wanted to see a doctor, because I wished a doctor could examine me. So I came to EC, because I know I get doctors at the hospital.’ (Participant 2, female, 60 years)

Linked with expectations around examination was a minor theme of poor explanation. This was the case for the clinical nurse practitioners as well as the doctors.

‘The only thing they [*sister*] do for you is to take your arm, take your blood pressure. … There wasn’t any further verbal interaction between us.’ (Participant 12, male, 27 years)

‘He [*doctor*] could have done it a little better … and at least said what the problem was, why it is that way. He didn’t explain to me … a man needs to know what the matter is.’ (Participant 6, male, 39 years)

‘You can’t say maybe it’s an infection. So you can’t be satisfied about that because you are not sure what happened to you. … I prefer to get the clear explanation. If there is something wrong to me, don’t be scared, just tell me … you can tell me straight.’ (Participant 20, female, 35 years)

### Uncertain follow-up

It was apparent that most participants received informal verbal safety netting by health care workers, stating that they should ‘come back’ if symptoms do not improve. Typically no follow-up date or next appropriate health-seeking behaviour was advocated. Participants volunteered this as their motivation to self-refer to the hospital EC:

‘She [*sister*] just told me I must take the pills and antibiotics until they are finished. If it is finished and I don’t feel better, then I must come back … but look, I can’t go back there again.’ (Participant 18, female, 57 years)

‘Let’s say doctor gives me this medication, after this medication then you tell me if you don’t feel better, then come back. … They didn’t say that word, so that is why I prefer to go myself to the hospital so that I can feel better.’ (Participant 20, female, 35 years)

Participant 10, who is a regular user of both the local clinic and the EC viewed this as the reason for repeated EC visit stating:

‘I believe if you have a need, you must just come to the EC. … That is basically what it is, you know. There is no follow-up for your problem … which actually is, now that I think about it, a very important aspect.’ (Participant 10, male, 60 years)

Participants presenting to a chosen health establishment did not necessarily consider the health system structure in terms of levels of care or referral pathways, but often acted on preconceived perceptions or out of uncertainty. This was fuelled by the minor theme of doubting whether it would help to go back to the point of their first consultation.

‘There wasn’t another option, because going to the clinic would not benefit me … because the clinic would send me here [*EC*]. They [*PHC*] would have said, go to the hospital, cause see there is nothing we can do for you.’ (Participant 8, male, 63 years)

‘I thought they [*PHC*] would tell me to come back tomorrow, that’s why I doubted going there [*PHC*]. … So I didn’t have a choice where to go to, but at George hospital I know they will be able to help me.’ (Participant 4, male, 48 years)

Participants’ perception of the referral structure within the health system sometimes seemed reversed from how the health system saw itself, first presenting to the EC, in hope of a referral to the clinic:

‘I would have gone to the clinic, but I first wanted to come here [*EC*] so that the doctor can refer me to go to that clinic.’ (Participant 7, male, 49 years)

### I did not know whether they were going to help me

The participants named long waiting times, the ‘number system’, staff shortages, availability of a doctor, pharmacy availability at the primary clinics and financial constraints as limiting factors to health care access. This influenced their health-seeking behaviour directly. Although frustrated by long waiting periods, participants seemed understanding to the limited staff attending to patients.

‘They first told me that there isn’t any more numbers. The clinic doesn’t have enough staff to help everybody.’ (Participant 1, female, 20 years)

‘At the day hospital you sit and wait the whole day.’ (Participant 4, male, 48 years)

‘When I got there [*PHC*] on Tuesday, it was not the day the pharmacy was open.’ (Participant 13, male, 35 years)

This uncertainty whether they will be attended to as a major theme, or told to ‘come back tomorrow’ that emerged as a minor theme, drove them away from the primary clinics.

‘Yes there is an uncertainty because sometimes, by the afternoon, they say they can’t help you and you need to come back tomorrow.… It did feel very strange, because I didn’t know whether they were going to help me.’ (Participant 10, male, 60 years)

‘That one [*sister*] is going to tell you, you must come back another day. You must make a date again … but you were already there. Just think, you must walk there again.’ (Participant 3, female, 53 years)

Doctors are only available on certain days and by appointment only at the PHC, which is usually scheduled months in advance. Participant 2 shared her frustration:

‘You must sit there from the morning, but if you need to see the doctor they [*sisters*] tell you you must make an appointment. You are deadly sick, you must see a doctor, you must make an appointment – you won’t be able to see a doctor now.’ (Participant 2, female, 60 years)

This was influenced further by financial limitations of the participants which played a large role in access to transport and seeking health care. Proximity to a health establishment determined access as explained by participant 20: ‘I just go to the place that I’m close to’.

Transportation is costly and some participants utilised the ambulance services as free transport:

‘Transport is expensive for us, we don’t have the money to pay transport … so I decided we must try the ambulance again.’ (Participant 11, male, 39 years)

The uncertainty of being helped at the clinic made some participants access a private GP, but still was left dissatisfied, and because of financial constraints were forced to access further public health care.

‘So I kind of need someone to sort it out and clearly my GP couldn’t do it because he needs more and that means more money and I can’t afford that right now.’ (Participant 19, female, 38 years)

### They told me I must rather go to the hospital

Family members, friends, employers and even health care providers tended to show more confidence in health care access and management at the hospital EC as opposed to public or private PHC.

‘I told my boss that I didn’t feel right and I can’t work like this. So he told then he must take me to the hospital. Then he took me to hospital.’ (Participant 16, male, 20 years)

‘Then she [*my mother*] asked me if I wanted to go to hospital and I said yes. Then she said okay she‘ll phone the ambulance.’ (Participant 11, male, 39 years)

Health care providers at the clinics as well as the GPs encouraged hospital EC preference. Participant 10 reflected on his recent clinic visit: ‘Like today I was at the clinic, then they told me I must rather go to the hospital … because they can’t really do anything for me there [*PHC*]’.

Participant 20 explained; ‘because I didn’t have medical aid, he [*GP*] suggested that I come here [*EC*]’.

The health system structure promotes access to the EC by ambulances transporting all patients to the EC directly.

‘Then she [*mother*] phoned the ambulance. They came to fetch me and brought me here [*EC*].’ (Participant 16, male, 20 years)

A number of themes that emerged were supported by this response:

‘I would rather come here [*EC*], because I don’t know if the clinic would be able to help me the same. The clinic is good, but they don’t have the resources.… Here the doctors can take X-rays. They can explain to me what is wrong with my lungs. I would rather decide to come here [*EC*], because I don’t get to see doctor every time at the clinic.’ (Participant 19, female, 38 years)

Access of care was understood and defined from the patient’s experience, and not from an understanding of the health system structure around appointments, triage criteria and referral pathways.

In contrast, this regular user of her PHC clinic and the hospital EC, had the following viewpoint:

‘There [*clinic*] was a nice attitude, every time I go there it is a good attitude … even the doctor gives me a hug … I prefer my clinic doctor.’ (Participant 14, female, 49 years)

‘They [*the doctors*] are very friendly. The hospital and the clinic, I don’t separate them. … They are one, and they are one with me.’ (Participant 14, female, 49 years)

## Discussion

The main reasons for seeking a second consultation were persistence of symptoms, particularly pain, closely associated with the need to understand the cause of the pain. This is in keeping with previous research as the main reason for unscheduled return visits to the emergency department being symptom related with inadequate pain management proven to be a significant problem.^1,7,8,9^ Milbrett identified that six of seven major reasons to visit an EC as having some relationship to pain, and White described pain-related diagnoses to be a major reason for unscheduled returns.^2,4^ Overdramatisation of pain, sometimes to the extreme of fear of death, raises the question of how well health care workers are addressing the presumed underlying physical or psycho-social contributing factors, and how this can be managed better.

**FIGURE 1:**
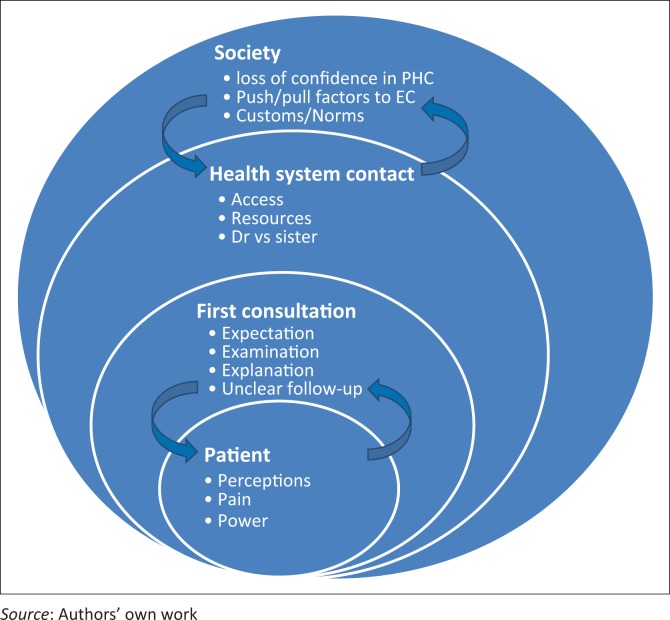
The ripple effect: Conceptual framework of issues and themes.

The reasons for a second consultation for the same complaint at the EC can be understood as issues relating to the patient, the first consultation, the health system and society. These issues cannot be analysed as separate entities but should rather be seen as a ripple effect starting at the patient, constantly interlinking with broader issues (Figure 1).

Patients, at the centre of the consultation, had preconceived perceptions about the severity of their illness, their preference for health access and possible treatment outcomes. Patients often viewed their complaints as more serious compared to the perceived opinions of health care workers, similar to findings in other settings.^3^ These perceptions were influenced by their beliefs, fears and prior experiences, as well as those of family and community members.^26^ Kleinman makes a clear distinction between ‘disease’, which is the usual focus of the health care workers, and ‘illness’, which is the patient’s experience of their symptoms. Herein lies a big assumption, namely that patient and health care worker find common ground during the consultation. We found the patients’ illness experiences were strongly influenced by the initial consultation. The lack of diagnostic certainty and a clear explanation were the biggest shortcomings in the initial consultation and served as internal motivation to seek a second consultation.^13,14,15^ The urge to understand and take more ownership of their diseases and illness behaviour was an important missed opportunity in the first consultation.

It has been shown that patients want a patient-centred approach. They expect health care practitioners to explore their main reason for the visit, their concerns, seeking an integrated understanding of the whole person and find common ground on what the problem is and mutually agree on management.^27^ Stewart, widely regarded as the pioneer of patient-centred care, agrees that patients should be the ones to define patient-centred care.^16^ From the health system point of view, it has been shown that patient-centred care is associated with reduced health care utilisation, reduced referral for special investigations and improved health outcomes.^28,29^

Family medicine worldwide and also in South Africa emphasises the importance of the bio-psycho-social approach to patient care in order to understand the patient’s illness experience, which has proven to be essential for accurate diagnosis, health outcomes and humane care.^30,31^ This is supported by the HealthCare 2030 Plan of the Western Cape Department of Health, which lists person-centred quality of care as the first principle in their vision statement.^21^ The holistic approach to the patient consultation incorporated into a shared management plan to improve health outcomes is stressed in policy but is not easily practised. This may be a big reason for many repeat consultations and failed patient expectations.

Access to emergency health care is a constitutional right in South Africa and health care providers or establishments may not refuse a person emergency care.^32^ This encourages and almost entrenches a sense of entitlement in the community to present to the hospital EC, especially if there is a perception that their symptoms are severe and they may not be sufficiently managed at a primary care facility.^5^ Paradoxically, we found that patients felt disempowered to access care as they expected in terms of investigations and a comprehensive, shared management plan. Participants had to ‘find the way’ of least resistance and personal cost for their expectations to be met. This translated into phoning an ambulance and going to the facility with the perceived least risk of failed expectations, which usually meant the EC.

The South African public primary health care service was designed to be run by clinical nurse practitioners as the first contact health care providers. This is often contradicted by the way society understands it, for example advocating to ‘see a doctor’ in case of illness. Expecting a doctor as the preferred health care provider, together with the consumerist world view of the need for special investigations to confirm a diagnosis, led to a negative appraisal of participants’ first consultation.^6^ We found the primary health care providers used the lack of resources as a reason to justify their diagnostic uncertainty. This reduced the credibility of primary care’s clinical diagnostic skills and a loss of confidence in primary health care in the minds of the patients.^3^ Dell’s study conducted at George hospital resonated this loss of confidence in PHC by stating the three main reasons for patients to attend the EC to be that the clinical treatment was not helping, they perceive hospital treatment to be superior and the lack of PHC services after hours.^5^ Being told to come back ‘tomorrow’ or ‘another day’ added to the participants’ uncertainty and was not perceived as sufficient safety netting in a comprehensive management plan.

The reasoning behind the pursuit of health care in most of the interviews was usually not a logical consideration of the health system and its appropriate entry point for a specific clinical complaint, but rather a response to a ‘socially shared custom’, as described by Beache and Guell.^3^ Health system factors enhanced and encouraged this custom. The habitual use of the hospital EC is not only appealing because of convenience, accessibility, staff and resource availability, but especially because of the likelihood of being attended to by a doctor, with the expectations of an examination and access to some investigations.^3,5^

In order for the primary health care service and the primary care component of hospital ECs to meet the expectations of patients and fully implement the holistic patient-centred model that is envisaged, it is important that consultations follow the bio-psych-social approach, together with a physical examination and a shared decision plan that clearly addresses the patient’s complaints, especially those relating to the experience of pain. Without this, patients’ perceptions and expectations of primary care will continue to be disappointed, with the default of access to hospital as perceived higher quality of care.^6^

## Conclusion

The aim of this study was to explore patients’ experience of their first consultation and any shortcomings that led to seeking an early second consultation at a regional hospital EC. The main themes included shortcomings in effective evaluation and management of pain, diagnostic uncertainty including poor explanation, poor examination, uncertain access, uncertain follow-up and societal encouragement to utilise the hospital EC.

Areas of possible intervention include exploring pain as a presenting symptom of the illness experience, and promoting competence in addressing physical and psychological causative factors within a patient-centred approach for all health staff, especially primary care services.
